# How does chronic unpredictable mild stress affect the number of mature oocytes? An experimental study

**DOI:** 10.18502/ijrm.v23i4.18782

**Published:** 2025-06-10

**Authors:** Alifia Candra Puriastuti, Margarita Maria Maramis, Jimmy Yanuar Annas, Reny I'tishom, Purwo Srirejeki, Sulistiawati

**Affiliations:** ^1^Midwifery Department, Faculty of Medicine, State University of Malang, Jalan Semarang No. 5, Malang, Indonesia.; ^2^Reproductive Health Science, Postgraduate Programme, Medical Faculty, Jalan Prof. Dr. Moestopo No. 47, Universitas Airlangga Surabaya, Indonesia.; ^3^Division of Biological Psychiatry and Neuroscience, Faculty of Medicine Airlangga University, Medical Faculty, Jalan Prof. Dr. Moestopo No. 47, Universitas Airlangga, Surabaya, Indonesia.; ^4^Department of Obstetrics and Gynecology, Medical Faculty, Universitas Airlangga, Jalan Prof. Dr. Moestopo No. 47, Surabaya, Indonesia.; ^5^Department of Biomedical Science, Medical Faculty, Universitas Airlangga, Jalan Prof. Dr. Moestopo No. 47, Surabaya, Indonesia.; ^6^Department of Medical Physiology, Medical Faculty, Universitas Airlangga, Jalan Prof. Dr. Moestopo No. 47, Surabaya, Indonesia.; ^7^Department of of IKM-KP, Medical Faculty, Universitas Airlangga, Jalan Prof. Dr. Moestopo No. 47, Surabaya, Indonesia.

**Keywords:** Chronic stress, Corticosterone, Oocyte.

## Abstract

**Background:**

The chronic unpredictable mild stress (CUMS) model involves applying mild stressors over a prolonged period, that can induce oxidative stress. Oxidative stress disrupts normal cellular functions, leading to reduced survival of antral follicles through increased glucocorticoids.

**Objective:**

This study aims to identify the effect of stress on the regulation of glucocorticoid hormones and its impact on impaired antral follicles. Specifically, it focuses on the number of antral follicles using the CUMS model in female rats.

**Materials and Methods:**

16 female Wistar rats (5–6 months, 300–350 gr) were divided into 2 groups (n = 8/each), the control and the CUMS model. 24 hr after the last treatment, they were eter euthanized, a blood sample was taken from the intracardial to measure corticosteroid levels using the enzyme-linked immunoassay (ELISA) method, ovarian preparations were made, and then the histological sections were observed.

**Results:**

After 22 days of CUMS, a significant difference was observed in corticosterone levels (p = 0.03), but no significant difference was observed in the number of antral follicles between the 2 groups (p = 0.57). However, histological analysis indicated substantial differences. The control group's ovaries exhibited a higher proportion of healthy antral follicles.

**Conclusion:**

CUMS increases glucocorticoids, which in turn causes a decrease in the number of antral follicles. This happens through 2 mechanisms: suppression of gonadotrophin-releasing hormone and direct effects on the ovaries that elevate granulosa cell apoptosis and follicular atresia, ultimately leading to a reduction in antral follicles.

## 1. Introduction

The chronic unpredictable mild stress (CUMS) model, characterised by the application of mild stressors over an extended period, mimics psychological stressors that can lead to various health problems, including reproductive dysfunction (1). This model has been used extensively to study the effects of stress on the endocrine system and reproductive health, revealing that chronic stress can induce oxidative stress and systemic inflammation, both of which impair ovarian function (2, 3). Oxidative stress disrupts normal cellular functions, leading to decreased oocyte quality and reduced survival of antral follicles (4). Antral follicles, which contain an antrum filled with follicular fluid, are products of oocytes and granulosa cells (5, 6). The development of antral follicles is influenced by estrogen and follicle-stimulating hormone (FSH) levels; these follicles have the potential to release preovulatory oocytes (7).

Sustained stress can impair folliculogenesis due to several factors, including disruption of the hypothalamic-pituitary-ovarian (HPO) axis (8). Oxidative stress is linked to increased suppression of gonadotropin-releasing hormone (GnRH) and dysfunction in folliculogenesis (7). Folliculogenesis, the process of follicular growth and development, relies on the collaboration of 3 types of follicular cells: oocytes, granulosa cells, and theca cells (9).

Moreover, excessive activity of corticotropin-releasing hormone (CRH) in the hypothalamus in response to stress leads to the adrenal glands producing glucocorticoid hormones, such as corticosterone. Cortisol plays a key role in various cellular events, including modulation of apoptosis, and the ovary is one of the target organs of cortisol (6, 10). Increased serum cortisol in response to chronic stress can impair reproductive function by affecting apoptosis and inhibiting the proliferation of granulosa cells. Cortisol, being a hydrophobic molecule, can penetrate the plasma membrane of target organs directly (11). Elevated cortisol levels also indirectly interfere with the GnRH pulse, subsequently reducing the production of gonadotropin hormone in the anterior pituitary (12, 13).

CUMS can alter the number of antral follicles primarily due to granulosa cell apoptosis, which is often mediated by hormonal imbalances, particularly involving FSH and its receptors (14). In the context of chronic stress, downregulation of FSH receptors on granulosa cells has been observed, making these cells more susceptible to apoptotic signals and affecting the follicle as a whole (15). Furthermore, chronic stressful environments can disrupt normal signalling pathways that promote the survival and proliferation of granulosa cells, which exacerbates follicular atresia (16). Prolonged exposure to stressors can lead to a state where granulosa cells become less responsive to growth factors and hormones, resulting in a diminished capacity for these cells to develop into antral follicles (17).

This study aims to identify the effects of stress on glucocorticoid hormone regulation and impaired folliculogenesis, by examining its impact on the number of mature follicles or antral follicles, utilizing the CUMS model in female rats (*Rattus norvegicus *[R. norvegicus]).

## 2. Materials and Methods

The experimental study design used control groups, which were positive control (18). The positive control was selected to compare of the mature oocytes between the control group that did not receive CUMS and the CUMS model groups that did receive CUMS. This research was conducted at the end of 2018 in Surabaya City.

### Animal models

The sample consisted of 16 female Wistar rats (5–6 months, 300–350 gr) (19), and having already given birth, indicating their viability. The rats were in good condition, exhibiting shiny and clean fur, fast movements, and normal demeanor. Pregnant or nursing rats, as well as any female rats that became ill or died during data collection, were excluded from the study.

The rats were adapted and acclimatized for 7 days in the Animal Center Laboratory, Faculty of Science and Technology, Airlangga University, Surabaya, Indonesia. They were housed in cages, each containing 2–3 rats in a quiet, calm, and well-ventilated study room. The environmental conditions included a room temperature of 25–28 C, relative humidity ranging from 40–70% and a 12-hr light/dark cycle, each rat was provided with 5 gr of food and 10 mm of water per day (19).

### Sample size

On 8 ${}^{\text{th}}$ day, 16 rats were divided into 2 groups the control group (n = 8) and the CUMS model groups (n = 8). Daily maintenance was conducted in both groups; the key difference being that the control group did not receive CUMS, while the CUMS group received CUMS treatment for 22 days.

### CUMS protocol

The CUMS approach was utilized in this investigation to induce chronic stress. A minimum of 3 wk is required between the administration of various therapies as stressors ensure they are comparable to regular stress but not excessively strenuous (2). Consequently, the treatment duration was aligned with the estrous cycle of R. norvegicus with the CUMS method being administered for 22 days as shown in table I (2, 20, 21) to coincide with the proestrus phase during surgery. To mitigate the adverse effects of acute stress, rats were euthanized 24 hr after the last treatment.

### Ensure the estrous cycle

On the 8 ${}^{\text{th}}$ and 10 ${}^{\text{th}}$ days, estrus synchronization was performed by intraperitoneally injecting prostaglandin F2alpha (PGF2, LutaprostⓇ 250, Agrivet, USA) at a dose of 25 g/g BW (18). The purpose of PGF2 injection is to synchronize estrous, which aims to balance the estrous phase of all samples that were in the preovulation phase until the time of termination (22–24).

### Ovarian tissue sampling

The tissue samples of the right and left ovaries were obtained through abdominal dissection and stored in 10% formalin for fixation. After fixation, the tissues were sliced to a thickness of 0.5 cm, dehydrated, and then embedded in paraffin blocks. These blocks were sliced into sections with a 5–7 µm thickness, which were mounted on glass slides. The sections were stained using hematoxylin-eosin preparations (18). A histological examination was performed using a light microscope, and the results obtained were recorded photographically using the software. 2 examiners carried out the slide examination, and the average was calculated. Photographs of the selected preparation were taken to represent each group, ensuring that all images were captured at the same magnification (x100, 10 field of view) (18).

### Sampling of blood and serum hormone assays

To prevent the consequences of acute stress, rats were euthanised with an excessive dose of ether 24 hr after their last treatment. The excessive eter euthanized method was chosen over the cervical spine dislocation because the blood sample was to be taken from the intracardial. The serum and other blood components were then separated by storing them in a marked test tube for 1 hr. Cold centrifugation was performed for 10–15 min at 3000 rpm. The separated serum was transferred into a labelled eppendorf tube and tested using ELISA method (18). This stage was conducted by laboratory staff at the Institute of Tropical Diseases, Airlangga University, Surabaya, Indonesia.

**Table 1:** The stressor in 22 days

**Day of treatment**	**Stressor (time)**	**Day of treatment**	**Stressor (time)**
**1**	Overcrowding or placed in one cage containing 5 rats (24 hr)	**12**	Immobilize with wire mesh restrainer (2 hr)
**2**	Isolation in a narrow and dark space (24 hr)	**13**	Tie the tail with thread (1 hr)
**3**	Tie the tail with thread (1 hr)	**14**	Isolation in a narrow and dark space (24 hr)
**4**	The cage is tilted 45 (5 hr)	**15**	The tail is pierced with a needle ± 2.5 cm long (1 hr)
**5**	Immobilize with wire mesh restrainer (2 hr)	**16**	Immobilize with wire mesh restrainer (2 hr)
**6**	Swimming in cold water 4 C (3 min)	**17**	Exposure to noise and loud sounds (85–90 dB) (3 hr)
**7**	Without stressor	**18**	Exposure to bright light of 300–400 lux (4 watts) is carried out twice a day (each 45 min)
**8**	Overcrowding or placed in one cage containing 5 rats (24 hr)	**19**	Overcrowding or placed in one cage containing 5 rats (24 hr)
**9**	Exposure to bright light of 300–400 lux (4 watts) is carried out twice a day (each 45 min)	**20**	Swimming in cold water 4 C (3 min)
**10**	Exposure to noise and loud sounds (85–90 dB) (3 hr)	**21**	The tail is pierced with a needle ± 2.5 cm long (1 hr)
**11**	Without stressor	**22**	Swimming in cold water 4 C (3 min)

### Ethical Considerations

The CUMS protocol and animal experimentation were reviewed and approved by Ethical Review Committee of Faculty of Veterinary Medicine, Universitas Airlangga Animal Care and Use Committee (ACUC), Universitas Airlangga, Indonesia (Code: 2.KEH.54.03.2018).

### Statistical Analysis

The data were analyzed using the SPSS statistical software package version 18.0 (SPSS, Inc., Chicago, IL). Initially, the data was normalized using the Shapiro-Wilk tests, followed by the Independent t test.

## 3. Results

We conducted vaginal swab examination before injection PGF2. Vaginal swab results indicated that the female R. norvegicus was in the preovulatory reproduction phase.

### Effect of CUMS on increased corticosterone levels

The results of blood tests using the ELISA method showed the effect of CUMS on increasing the corticosterone hormone, namely with the following results:

Table II displays the corticosterone levels (ng/mL) in R. norvegicus (rats) measured 24 hr following 22 days of CUMS treatment. The table demonstrated that median corticosterone levels were higher in the CUMS model (26.61 ng/mL) than in the control group (15.85 ng/mL), indicating that corticosterone levels increased overall after CUMS exposure. The minimum corticosterone levels were significantly higher in the CUMS model group (17.28 ng/mL) than in the control group (7.07 ng/mL), indicating an increase in baseline corticosterone levels. The maximum corticosterone levels increased significantly in the CUMS model group (82.40 ng/mL) compared to the control group (20.34 ng/mL), indicating a considerable stress response.

The CUMS model group had significantly higher mean corticosterone levels (33.56 ± 21.28 ng/mL) than the control group (13.36 ± 4.31 ng/mL), indicating increased stress-induced corticosterone production. A p = 0.03 indicates a statistically significant difference between the groups, implying that chronic stress has a significant effect on corticosterone levels.

### Effect of CUMS on the number of antral follicles

The effect of CUMS on the number of antral follicles can be observed histopathologically, with the following results:

Table II shows the number of antral follicles in R. norvegicus after 22 days of CUMS administration. The median follicle count was the same in both groups (2.00), implying no significant variation in central tendency. The minimum follicle count was 0 in both groups, indicating that some animals lacked antral follicles; however, the maximum follicle CUMS model group (4) than in the control group (6), possibly indicating a stress-related drop in follicle numbers.

The CUMS model group had slightly lower mean ± SD follicle counts (2.30 ± 1.06) than the control group (2.67 ± 1.66), suggesting a modest decline in follicle numbers after CUMS. The p = 0.57 indicates that the difference between groups is not statistically significant. This suggests that chronic stress exposure did not significantly reduce the number of antral follicles in this experimental scenario.

**Figure 1:**
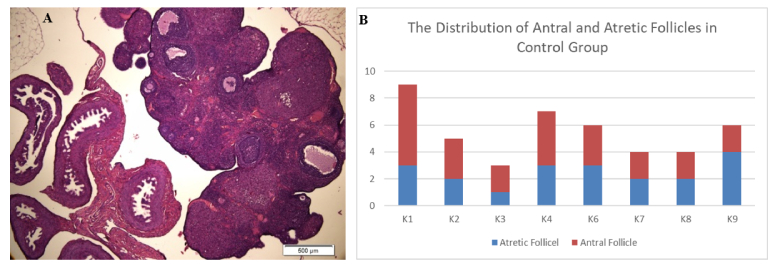
A) Histology of ovarian follicles in normal control group, B) Distribution of antral and atretic follicle numbers in normal control group.

However, the ovarian histology of the control group exhibited better stages of folliculogenesis, including primary follicles, secondary follicles, antral follicles, and preovulatory follicles. This finding was supported by a higher distribution of antral follicles in the control group compared to atretic follicles in the CUMS model (Figure 1).

**Figure 2:**
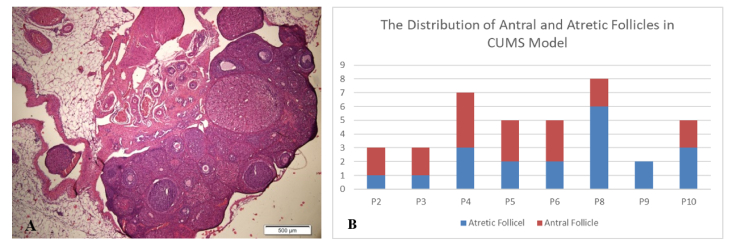
A) Histology of ovarian follicles in CUMS model group, B) Distribution of antral and atretic follicle numbers in CUMS model group. CUMS: Chronic unpredictable mild stress.

When antral follicles were present in the CUMS model, the histology displayed larger antrum size than those observed in the control group (Figure 2). The size of the antrum appeared disproportionate to the thickness of the granulosa cell layer, and in some instances, oocyte cells were absent from in the antral follicles in the CUMS group.

**Table 2:** Corticosterone levels and antral follicle count in R. norvegicus after 22 days of CUMS

**Parameters**	**Control (n = 8)**	**CUMS model (n = 8)**	**P-value**
**Corticosterone level (ng/mL)**	13.36 ± 4.31 15.85 (7.07–20.34)	33.56 ± 21.28 26.61 (17.28–82.40)	0.03
**Antral follicle count**	2.67 ± 1.66 2.00 (0–6)	2.30 ± 1.06 2.00 (0–4)	0.57
Data presented as Mean ± SD, median (min-max), independent t test. CUMS: Chronic unpredictable mild stress			

## 4. Discussion

This study's limitation was due to the limited literature available on CUMS and its relation to reproductive health conditions. Hence, the authors use a lot of literature related to stress in general.

### Effect of on increased corticosterone levels

A significant difference in corticosterone levels between the CUMS model group and control group, with the CUMS model group exhibiting higher levels. Corticosterone is the primary glucocorticoid hormone in rodents, similar to how cortisol functions in humans (25). A prior study showed that corticosterone acts as a biomarker linked to chronic adaptation, whereas cortisol reflects more reflective of severe acute stress (26).

Corticosterone and cortisol are glucocorticoids that mediate the systemic stress response. These hormones are produced by the adrenal glands as part of the body's homeostatic response to stressors (8). The secretion of glucocorticoid is stimulated by adrenocorticotropin hormone, which is secreted by the anterior pituitary. The release of adrenocorticotropin hormone is, in turn, stimulated by corticotrophin releasing hormone (CRH) from the hypothalamus during stress conditions (27).

The elevated corticosterone levels observed in the CUMS group align with López's research, which reported increased corticosterone levels after 20 days of CUMS exposure (2). The CUMS method was utilized to stimulate various stressors, including daily mild stressors. Many individuals encounter minor stress without realizing its impact on their health. If these daily pressures remain unaddressed, it can lead to depression (2, 20), affecting overall physiological functions, including reproductive health.

Glucocorticoids play a crucial role in various cellular activities, such as regulating apoptosis, responding to stress response and managing lipid and glucose metabolism, as well as mitochondrial function (10). Increased glucocorticoid release and synthesis in response to acute or chronic stress can compromise reproductive function in many animals. Research by Scarlet et al., explains that the ovaries are one of the primary targets of glucocorticoid (6).

Glucocorticoids disrupt normal ovulation through 2 mechanisms: first, by affecting the HPO axis connection and second, by directly impacting ovarian tissue within the follicle. Under normal circumstances, the HPO axis starts with the hypothalamus releasing GnRH. This process triggers the anterior pituitary to produce FSH and luteinizing hormone (LH), which are essential for follicular development (27). Glucocorticoids modulate the HPO axis by inhibiting GnRH release from the hypothalamus and disrupting the synthesis and release of gonadotropins (FSH and LH) from the pituitary gland. Elevated corticosterone levels during chronic stress do not provide negative feedback to the hypothalamus to reduce CRH production (8). Consequently, excess CRH is continuously produced, which further suppresses GnRH secretion (12).

Previous studies have shown that cortisol reduces LH and progesterone levels in granulosa cells of both rats and humans. Additionally, elevated glucocorticoid levels due to restraint stress have been found to impair oocyte development potential in ex vivo evaluations (28).

### Effect of CUMS on the number of antral follicles

The second mechanism of ovulatory dysfunction involves glucocorticoids flowing through the peripheral blood into ovarian tissue, where they bind to corticosterone-binding protein in the follicle. This binding stimulates various mechanisms, including direct effects on steroidogenesis that influence oocyte quality (10). Additionally, glucocorticoids affect ovarian physiology by regulating the function of granulosa cells, oocytes, cumulus cells, and luteal cells (28).

Histological analysis of the ovaries from CUMS model showed that the size of the antral follicles was larger than that in the control group. The size of the antral follicle is determined by the antrum, which contains follicular fluid, a product of plasma exudate, oocytes, and granulosa cells (29). The proliferation of follicular cells (both granulosa cells and theca cells) also plays a role in determining follicle size. Furthermore, the thickness of the cumulus cell layer, which is made up of granulosa cells surrounding the follicular antrum, appeared thinner in the CUMS model compared to the control group. This could suggest physiological atrophy of antral follicles with numerous granulosa cells undergoing apoptosis due to hormone disturbance or direct impacts (21, 30). In addition to the size of the antrum, which appears much larger, there is no oocyte as part of the follicle. In a healthy antral follicle, a variety of cell types-including oocytes, mural granulosa cells, cumulus cells, and a collagen-rich base that separates the follicle from the stromal compartment-should be present (29).

Granulosa cells are crucial for the normal progression of the female reproductive cycle (31). Any alteration in the granulosa cell cycle is believed to disrupt follicular growth and oocyte maturation (32). When homeostasis in granulosa cells is disturbed, it can result in suboptimal cell proliferation, preventing the formation of an optimal oocyte-cumulus complex layer.

If the granulosa cells, which are responsible for capturing FSH, become disrupted, the process of follicle growth is negatively impacted. The release of FSH decreases as GnRH secretion is suppressed by CRH. These 2 mechanisms can create a vicious circle contributing to the pathophysiology of granulosa cell proliferation. By the time the follicle reaches the antral phase, it cannot adequately support the needs of the oocyte and the steroidogenesis process. The condition of granulosa cells becomes increasingly unbalanced, and some may even undergo apoptosis. This extensive apoptosis of granulosa cells significantly impacts oocyte quality (31, 32). Research conducted by Regan et al. (32) has demonstrated that follicular atresia begins with granulosa cell apoptosis. Increased cortisol levels have been associated with heightened apoptosis in isolated mural granulosa cells, which correlates with elevated levels of Fas ligand. Subsequently, cumulus cells also become more sensitive to apoptosis under serum-starved conditions (28).

## 5. Conclusion

CUMS increases glucocorticoids, which in turn causes a decrease in the number of antral follicles. This happens through 2 mechanisms: suppression of gonadotrophin-releasing hormone and direct effects on the ovaries that elevate granulosa cell apoptosis and follicular atresia, ultimately leading to a reduction in antral follicles.

##  Data Availability

The original data of the current study can be available from the corresponding authors at a reasonable request.

##  Author Contributions

AC. Puriastuti is the main author in this research, who developed the idea and topic of the research. This research builds upon the CUMS method developed by MM. Maramis. The research design was guided by both MM. Maramis and JY. Annas. The data collection technique was implemented under the supervision of R. I'tishom. Additionally, sorting techniques, data analysis, and data writing were taught by P. Srirejeki and Sulistiawati.

##  Conflict of Interest

The authors declare that there is no conflict of interest.
